# Three‐dimensional view of microglia—amyloid plaque interactions

**DOI:** 10.1002/glia.24628

**Published:** 2024-10-22

**Authors:** Maria Gotkiewicz, Janne Capra, Pasi O. Miettinen, Teemu Natunen, Heikki Tanila

**Affiliations:** ^1^ University of Eastern Finland A.I. Virtanen Institute Kuopio Finland; ^2^ Institute of Biomedicine University of Eastern Finland Kuopio Finland

**Keywords:** 3D rendering, ApoE, confocal imaging, dense‐core plaque, transgenic mice

## Abstract

Recent gene expression studies have revealed about 10 different states of microglia, some of which are characteristic for Alzheimer‐like amyloid plaque pathology. However, it is not presently known how these translate into morphological features that would reflect microglia interaction with amyloid plaques. With optimized conditions for confocal microscopy in amyloid plaque forming APP/PS1 transgenic mice we reveal new details of how microglia processes interact with amyloid plaques. The microglia contacts differed drastically between purely diffuse plaque and those with a fibrillar core. We identified microglia that extend their enlarged processes through the diffuse shell of the amyloid plaques and cover the fibrillar plaque core with snowplow‐like expanded end‐feet. These end‐feet were filled with the lysosomal marker CD68, while both non‐fibrillar and fibrillar amyloid was found in perinuclear vesicles of some “snowplower” microglia. In the organized dense‐core plaques, we consistently saw a layer of Apolipoprotein E (ApoE) between the fibrillar core and the microglial end‐feet. ApoE covered also loose fibrillar amyloid and diffuse amyloid plaques that were about 10 μm or larger in diameter. These findings are compatible with both amyloid plaque phagocytosis and compaction by microglia. Further, they support a chemotactic role of ApoE for microglia contacts with amyloid plaques.

## Main Points

- Microglia interact differently with diffuse and fibrillar plaques.
- Microglia aim their enlarged processes through the diffuse amyloid layer to the fibrillar core and cover it.
- ApoE forms a layer between the plaque core and the microglia processes.

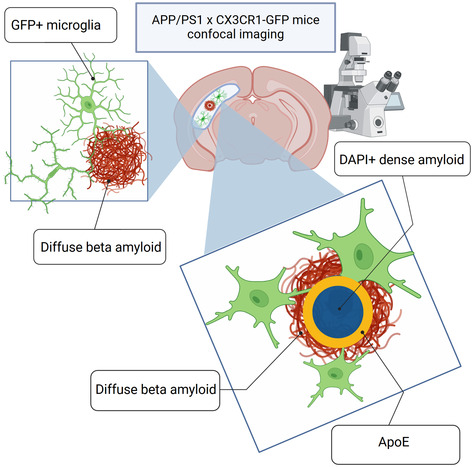

## 1 INTRODUCTION

Recent advancement in Alzheimer's disease (AD) research has brought up the central role of microglia in the core pathology, amyloid plaque formation and its impact on the surrounding neuropil. First, recently identified new gene variants influencing the AD risk have been genes mostly expressed in microglia (such as *ABI3*, *PLCγ2*, and *TREM2*; Sims et al., 2017) Second, single‐nuclei RNA‐sequence studies have identified up 10 different gene expression patterns of brain microglia, (Prater et al., 2023) which presumably reflect different types of microglia interactions with the neural tissue. Notably, one of these, so‐called disease‐associated microglia (DAM) is typically seen around amyloid plaques (Keren‐Shaul et al., 2017) and requires ApoE and TREM2 signaling to reach this activation state. However, there is much less data on how these various microglia gene expression patterns relate to changes in microglia morphology and expression of immunohistochemical markers.

Amyloid plaques are roughly divided into two types: diffuse and dense‐core ones (Zaletel et al., 2021). Diffuse plaques are composed of loosely packed amyloid fibrils forming a cloud, often without activated microglia cells around them, while dense‐core plaques attract microglial cells (Amin et al., 2023). However, it is not yet clear whether the way microglia interact with an amyloid plaque depends on the plaque type or composition.

Further, there are divided views on what is the primary action of microglia toward the amyloid plaques. In general, plaque‐associated microglia extend their processes toward the plaques and seemingly try to cover its surface, although the coverage is often incomplete (Condello et al., 2015; Mandrekar‐Colucci & Landreth, 2010). Some researchers believe that the main role of the microglia is to isolate the amyloid plaques and thereby to prevent the plaque growth and spread of toxic soluble Aβ oligomer into the surrounding neuropil (Ulland et al., 2017; Zhao et al., 2017). In contrast, others favor the idea that microglia first attempt to phagocytose the plaque amyloid and dispose of it by proteolytic degradation (Lee & Landreth, 2010).

A major hurdle in understanding the details of microglia—amyloid plaque interactions is the complex 3‐dimensional spherical structure of the amyloid plaques that with the surrounding cloud of microglia has a far bigger diameter than the ~5 μm of routine paraffin sections used in human neuropathology and many experimental studies. Even a 30 μm section may not contain an intact amyloid plaque in a transgenic mouse if the plaque is not located in the very center of the section. Therefore, most published studies show images with only part of the plaque associated microglia. With thicker sections, the antibody penetration may also become a problem, resulting in impaired resolution of microglia fine structure.

We approached the challenge of visualizing plaque associated microglia by combining several recent technical improvements compared to routine immunohistochemistry. First, we used amyloid plaque producing APP/PS1 transgenic mice cross‐bred with CX3CR1‐GFP mouse line with GFP selectively expressed in microglia (Condello et al., 2015). This endogenous fluorescence enabled strong and homogenous signal throughout the microglia including the thin processes. Third, we used a confocal microscope with the Airyscan module and an extensive z‐stack to obtain a 3D visualization of the plaque with the surrounding microglia, allowing us to separate microglia in contact with the plaque from those above or below the plaque. Finally, we utilized our recent observation that the standard nuclear DAPI staining acts like Thioflavin‐S or other nonimmunological amyloid plaque ligand (Mabrouk et al., 2022). Thus, one color channel could visualize the amyloid plaque core but also the nucleus of the surrounding microglia, thus helping to identify the microglia soma from its processes.

This approach helped us see some details in microglia—amyloid plaque interactions that have not been described before and help understand the complex role of microglia in AD pathology.

## 2 METHODS

### 2.1 Animals

Mice from a colony maintained in the animal facility of University of Eastern Finland in Kuopio, carrying mutated human APPs we and PSEN1dE9 transgenes (Jankowsky et al., 2004) were crossbred with mice carrying transgenic GFP protein in CX3CR1 receptor in microglia cells (strain CX3CR1‐GFP) (Jackson Laboratory, Bar Harbor, ME, USA, stock #005582). We will call this resulting line APdE9 × GFP line for brevity. Female offspring were used in this study. Mice were group housed in individually ventilated cages in controlled environment with light/dark cycle 12/12 h and ad libitum access to fresh water and normal mouse chow. The experiments were conducted in accordance with the EU guidelines and were approved by the Animal Experiment Board of Finland.

### 2.2 Tissue collection

Female mice were sacrificed at the age of 6 and 13 months. We used three + three (6 + 13 months) APdE9+ × GFP+ and two (13 months) APdE9+ × GFP‐ mice in the study. Under deep pentobarbital/chloralhydrate anesthesia (Equithesin) mice were transcardially perfused with ice cold saline. Brains were removed from the skull and split at the midline. Only the left hemisphere excluding the cerebellum and olfactory bulb was used. Thereafter, the tissue was immersion fixated in 4% PFA for 4 h and then transferred to 30% sucrose overnight for the dehydration of the tissue. Brains were stored in −20°C in antifreeze solution until further use. Tissue sectioning was done with a freezing slide microtome (Leica SM2000R, Wetzlar, Germany) into a series of 35–100–35 μm thick coronal sections.

### 2.3 Immunohistochemistry

For immunofluorescence brain staining we chose 100 or 35 μm coronal sections of the left hemisphere from APdE9+ × GFP+ or APdE9+ × GFP‐female mice at 6 and 13 months of age. The sections were taken from −20°C antifreeze storage and left at RT overnight, in dark, on a shaker table in Na_3_PO_4_ buffer (0.1 M, pH 7.6).

For the combinations of X34 + D54D2 and X34/ApoE/GFP we used the following staining protocol:

The next day, working solution of 0.05 or 0.01 mM X34 (Sigma Aldrich Cat. no SML 1954) was prepared in 60% PBS and 40% EtOH, at end brought to pH = 10. Sections were left to incubate for 1 h at RT/dark/shaker. Thereafter a brief washing with 60% PBS/40% EtOH solution was performed, followed by 3 × 5 min washing in 0.5 M TBS‐T pH 7.6. After this step, primary antibodies were applied.

For other immunostainings, the protocol was the following:

On the next day, sections of GFP+ mice were rinsed 3 × 5 min in TBS‐T (TBS + 0.5% TritonX, 0.5 M, pH 7.6) and stained free floating in RT overnight in dark on a shaker table with the following primary antibodies:

- Rabbit monoclonal anti‐human amyloid‐β 1:10000 (ref 08/20, D54D4 XPCR, Cell Signaling Technology, USA)
- Rat‐anti mouse CD68 (1:4000, cat. MCA1957 Bio‐Rad, USA). Before staining with the CD68 primary antibody the sections were blocked for 30 min in 10% NGS (Normal Goat Serum) in TBS‐T, followed by second round of 3 × 5 min washes in TBS‐T.
- Goat polyclonal anti‐human ApoE (1:8000, Anti‐Apolipoprotein E Goat pAb, ref. 178479, Merck, USA). ApoE staining together with D54D2 staining was done for 35 μm sections of GFP‐mice. Before incubating in the primary antibody, we performed antigen retrieval by heating sections in 0.05 M citrate solution at 85°C for 30 min. After cooling down in 0.1 M Na_3_PO_4_ buffer (0.1 M, pH 7.6) to RT, we performed triple wash in 0.5 M, pH 7.6 TBS‐T, and applied primary antibodies overnight at RT in dark on a shaker at the following concentrations: D54D2 1:10000, ApoE 1:8000.

Next day, sections were washed 3 × 5 min in 0.5 M pH 7.6 TBS‐T and the secondary antibody in dilution of 1:400 conjugated with corresponding Alexa‐Fluor 488 or 594 (Invitrogen, USA) was applied to sections for 2 h in RT in the dark on a shaker. After incubation, the last triple wash in TBS‐T 0.5 M, pH 7.6 was performed. Sections were mounted on gelatin coated glasses (Mendel‐Gläser, Thermo Scientific, USA) with VectaShield HardSet Antifade Mounting Medium with DAPI (Vector Laboratories, USA) and kept at +4°C until imaging. The DAPI in the medium worked also as staining for the fibrillar part of the amyloid plaques (Mabrouk et al., 2022).

For Thioflavin‐S alone the staining protocol was as follows:

After overnight wash in 0.1 M Na_3_PO_4_ buffer (pH 7.6) sections were mounted on a slide and dried overnight at +35°C. Thereafter, they were washed 2 × 5 min in dH20, followed by 20 min incubation in 1% solution of Thioflavine‐S in dH20 (cat. T‐1892, Sigma Aldrich, USA) in dark. After that, glass underwent sequence of washes: quick rinse 2× in dH2O, then 1 min each, in following order: 50% EtOH, 70% EtOH, 80% EtOH. Then for 2 min in following order: 96% EtOH, 100% EtOH, 0.1 M PB. After the last wash, glasses got mounted with mounting medium VectaShield HardSet Antifade Mounting Medium without DAPI (Vector Laboratories, USA) and kept at +4°C until imaging.

For the Thioflavin‐S coupled with D54D2 antibody, we followed this protocol:

Rabbit monoclonal anti‐human amyloid‐b 1:10000 (ref 08/20, D54D4 XPCR, Cell Signaling Technology, USA) staining was performed as described previously, including secondary antibody incubation. After last washing 3 × 5 min in 0.5 M TBS‐T pH 7.6, an 8 min incubation in 0.1% ThioflavineS in 0.5 M TBS‐T pH 7.6 (cat. T‐1892, Sigma Aldrich, USA) in dark was performed. After that, double rinsing for 1 min in 50% EtOH was done, followed by 5 and 1 min washing in Na_3_PO_4_ buffer (0.1 M, pH 7.6). As the last step, glasses got mounted as above.

### 2.4 Imaging

Imaging was performed at the Cell and Tissue Imaging Unit of University of Eastern Finland using a confocal microscope (LSM 800 with Airyscan module, 63×/ NA 1.4 oil immersion objective, ZEISS, Germany). A z‐stack of 20–70 with 0.5 μm increment was collected across selected amyloid plaques. The regions of interest were the molecular layer of the dentate gyrus and the stratum oriens of the CA1 and CA2 area of the hippocampus.

For the analysis, we imaged 2–3 brain sections per animal and aimed to collect 20–30 plaques per mouse. We took pictures of isolated microglia‐amyloid clusters to have a clear interpretation of microglia cells gathered around plaques not bigger than 21 μm of diameter.

For the imaging we used lasers lines as follows:

- GFP/Alexa Fluor 488: 488 nm laser line, excitation wavelength 493 nm, emission 517 nm, detection 450–585 nm
- Alexa Fluor 594: 561 nm laser line, excitation wavelength 560 nm, emission 618 nm, detection 580–700 nm

DAPI: 405 nm laser line, excitation wavelength 353 nm, emission 465 nm, detection 400–580 nm.

All channels were imaged separately using LSM800 Airyscan detector, with a gain of 850 V.

Primary 3D rendering was performed in ZEN 3.3 blue edition (ZEISS, Germany) and further chosen plaques were rendered in IMARIS x64 (ver 10.0.1, Oxford Instruments, UK) with MatLAB plugin IMARIS XTension Surface‐Surface Contact Area.

### 2.5 Analysis of microglia interactions with amyloid plaques

All analysis of microglia was based on clusters surrounding an isolated amyloid plaque with no satellite plaques. All GFP+ microglia with a visible soma and any kind of direct contact with the plaque were included. The microglia soma was distinguished from enlarged processes by the presence of a typical telephone dial‐like chromatin pattern. The 3D image stack was viewed at different angles to make a distinction between true microglia—plaque contact from overlap in the standard 2D view.

The type of microglia interaction with the amyloid plaque was classified as described in the Results section into four categories (scores) that constituted a continuum from the weakest to the strongest (1–4). The interaction type for each cluster of microglia was calculated as an average for all microglia in the cluster.

### 2.6 Statistical analysis

Statistical analysis was performed in IBM SPSS Statistics 27. Comparisons between three ordered categories were done with one‐way ANOVA, while Spearman rank correlations was used for data with six ordered categories. The limit for statistical significance was set to *p* = 0.05.

## 3 RESULTS

We imaged in total 61 amyloid plaques with the surrounding cluster of microglia in brain sections from three 13‐month‐old and 44 amyloid plaques in three 6‐month‐old APdE9+/GFP+ female mice using anti‐Aβ D54D2 staining. The plaque diameter ranged from 3 to 25 μm. We avoided larger plaques as they usually had satellite plaques and too many microglia around them to enable analysis of the contacts of a single microglia cell. We limited our analysis to the dentate molecular layer and CA1/CA2 stratum oriens that are almost free of neuronal nuclei that would block the view on microglia nuclei in the DAPI stained sections.

**FIGURE 1:**
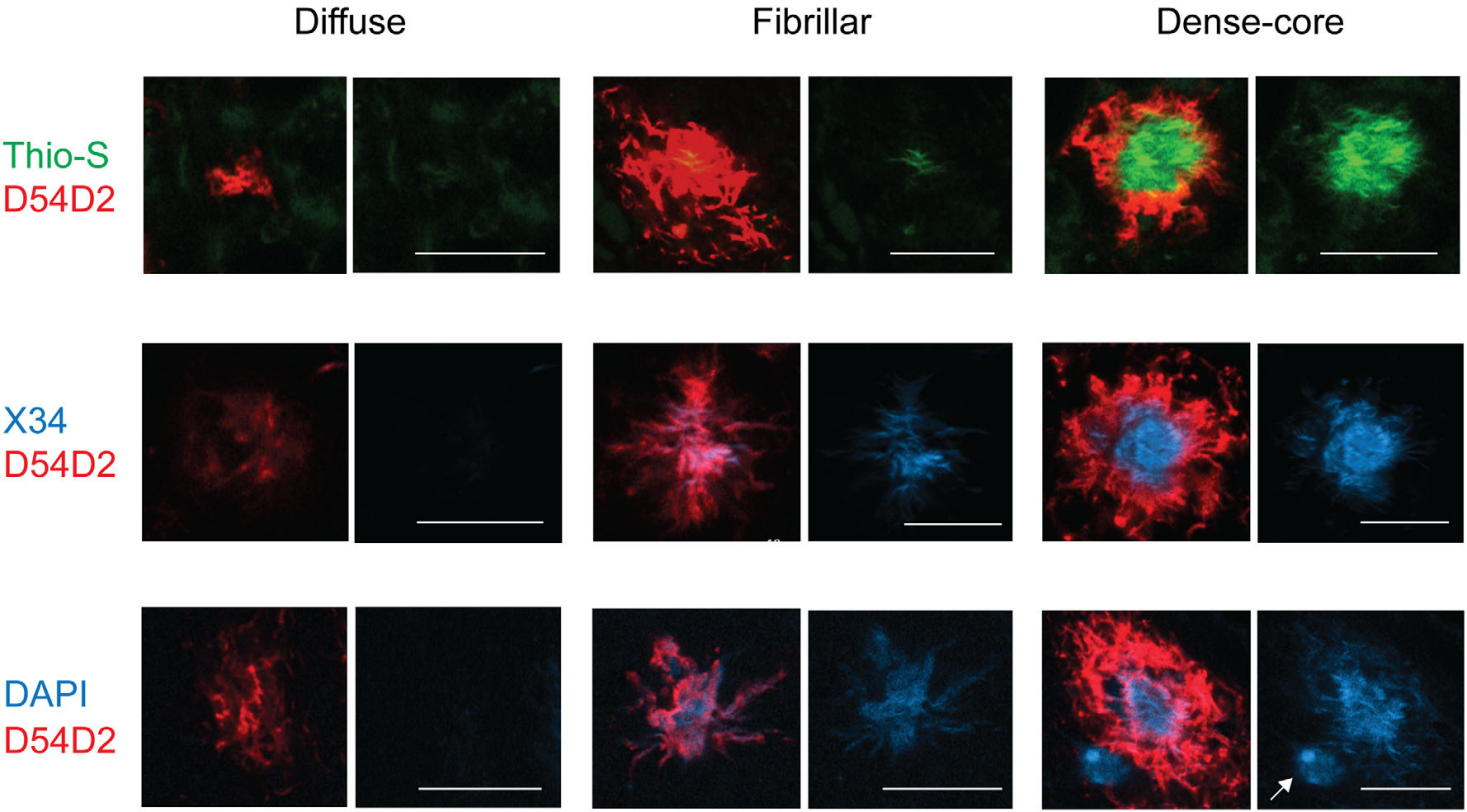
Composition of diffuse, fibrillar, and dense‐core amyloid plaques stained with three dyes binding to fibrillar amyloid, Thioflavin‐S, X34, and DAPI, and the N‐terminal anti‐Aβ D54D2 antibody. The *diffuse* plaques are well visible with anti‐Aβ D54D2 antibody but show no staining standing out from the background with Thio‐S, X34, or DAPI. The *fibrillar* plaques show a consistent signal in the plaque center that has fibrillar or cotton wool‐like texture radiating to all directions. Of the fibrillar amyloid dyes, X34 is the most avid to stain single fibril‐like structures. The *dense‐core* plaques display a clear round center that is surrounded by a D54D2+ cloud. Thio‐S stains the core very intensively with a sharp boundary. In contrast, X34 staining shows a ragged boundary between the core and surrounding fibrillar amyloid. DAPI staining, on the other hand, delineates the dense core with intensive staining and is surrounded by a less intense halo of fibrillar amyloid. Note that only DAPI stains the nuclear chromatin (white arrow). Scale bar = 10 μm.

By combining D54D2 immunostaining with DAPI staining we could differentiate amyloid plaques into three composition categories (Figure 1). All plaques showed strong D54D2 positivity but differed in their DAPI staining pattern as follows. (1) Diffuse plaques displayed no or only faint DAPI signal with no recognizable texture. (2) Fibrillar plaques had a clearly visible homogenous DAPI+ core with irregular shape and a fibrillar texture. (3) Dense‐core plaques showed a plum stone‐like bright DAPI+ center surrounded by a halo of less intense DAPI+ signal. To link the DAPI‐based amyloid plaque categorization with earlier work using other stainings (cf. Dickson & Vickers, 2001), we stained a subset of section also with Thioflavin‐S + D54D2 and X34 + D54D2 combinations. As shown in Figure 1, DAPI worked like the other two dyes selective for fibrillar amyloid and did not stain the smallest diffuse plaques. However, whereas Thioflavin‐S mostly stained the dense plaque core, X34 made less distinction between the core and the radiating fibrils. DAPI in turn stained the dense plaque core like Thioflavin‐S but also stained surrounding fibrillar amyloid but with lower intensity.

### 3.1 Amyloid plaque composition depends on the plaque size

**FIGURE 2:**
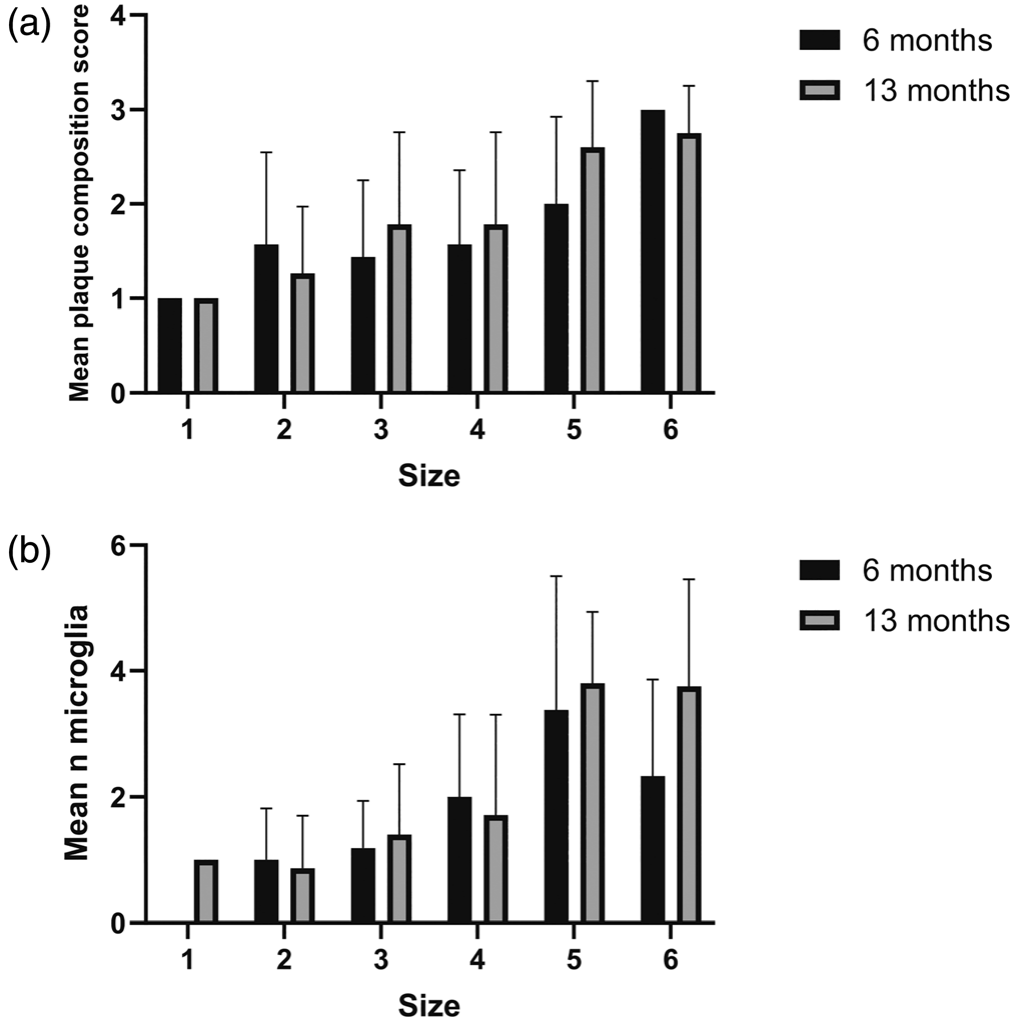
(a) Correlation between amyloid plaque size and amyloid plaque composition in 6‐ and 13‐month‐old APP/PS1 mice. Composition categories (1 = diffuse, 2 = fibrillar, 3 = dense‐core). There was a significant shift from diffuse toward dense‐core plaques at both 6 months (*R* = 0.35, *p* = 0.02, *n* = 42) and 13 months (*R* = 0.53, *p* < 0.001, *n* = 60) of age. (b) The mean number of microglia around an amyloid plaque depends on the plaque size. Size categories based on diameter (cat 1 = 3–5 μm, cat 2 = 6–7 μm, cat 3 = 8–10 μm, cat 4 = 11–15 μm, cat 5 = 16–20 μm, cat 6 > 20 μm). All sizes of plaques were rounded up to full numbers. For 6‐month‐old: *R* = 0.59, *p* < 0.001, *n* = 44, for 13‐months‐old: *R* = 0.57, *p* < 0.001, *n* = 61; Spearman correlation.

The three amyloid plaque composition categories appear as stages of a continuous process. In vivo imaging studies have demonstrated that the amyloid plaques also grow until they reach a critical size (Burgold et al., 2011). To assess whether these two trends were related to each other, we divided the amyloid plaque diameter into six size categories and correlated these with the three types of amyloid plaques (Figure 2a). There was indeed a significant correlation between the plaque size and the plaque composition category at both ages (6 months: *R* = 0.35, *p* = 0.02, *n* = 42; 13 months: *R* = 0.53, *p* < 0.001, *n* = 60; Spearman rho).

### 3.2 The number of microglia and their interaction with the amyloid plaque depend on the plaque size

As expected, the number of microglia in the cluster around an amyloid plaque grew with the plaque diameter at both ages (6 months: *R* = 0.59, *p* < 0.001, *n* = 44; 13 months: *R* = 0.57, *p* < 0.001, *n* = 61; Spearman rho), as show in Figure 2b.

**FIGURE 3:**
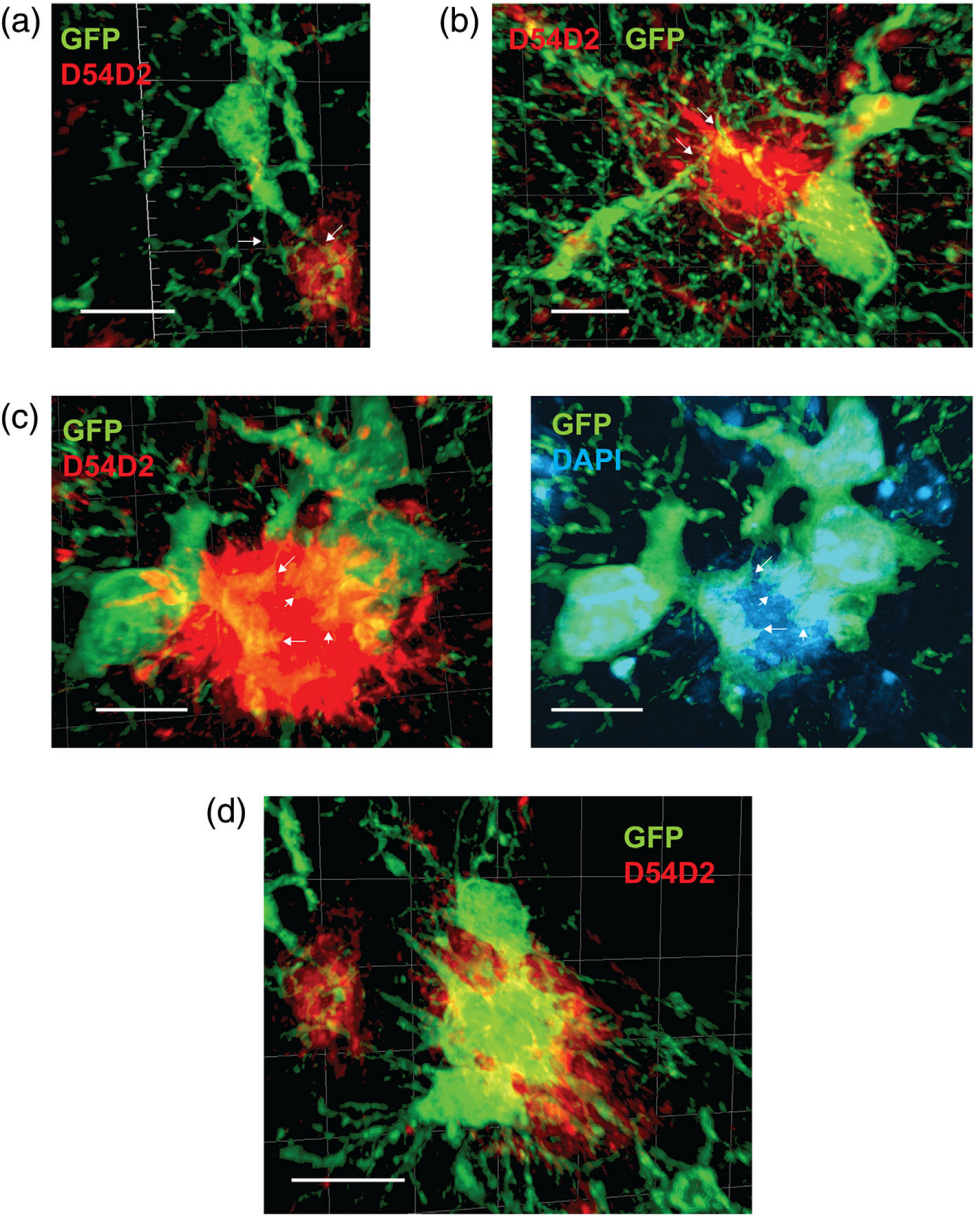
Examples of microglia–amyloid plaque interaction categories. (a) Category 1: the amyloid plaque is contacted by processes of a single microglia (arrows). (b) Category 2: the amyloid plaque is contacted by processes of two microglia (arrows). Note that the body of the microglia at 4 o'clock may seem to contact the plaque but a side view shows it is on a different z‐level. (c) Category 3: two microglia encroach into the amyloid plaque with enlarged processes (arrows and arrowheads). (d) Category 4: the microglial body is directly in contact with the plaque. Scale bar = 10 μm.

An interesting question is whether the way the microglia interact with the amyloid plaque also changes as the plaque grows. To this end, we identified four different categories of microglia—amyloid plaque interactions from weak to strong based on a previous classification (Jung et al., 2015) as follows: (1) amyloid plaque contacted by a single surveying microglial process; (2) Amyloid plaque contacted by various microglial processes; (3) Amyloid plaque enclosed by enlarged snowplow‐like microglial processes; (4) Amyloid plaque in direct contact with the microglial cell body. Examples of these interaction categories (1–4) as given in Figure 3.

**FIGURE 4:**
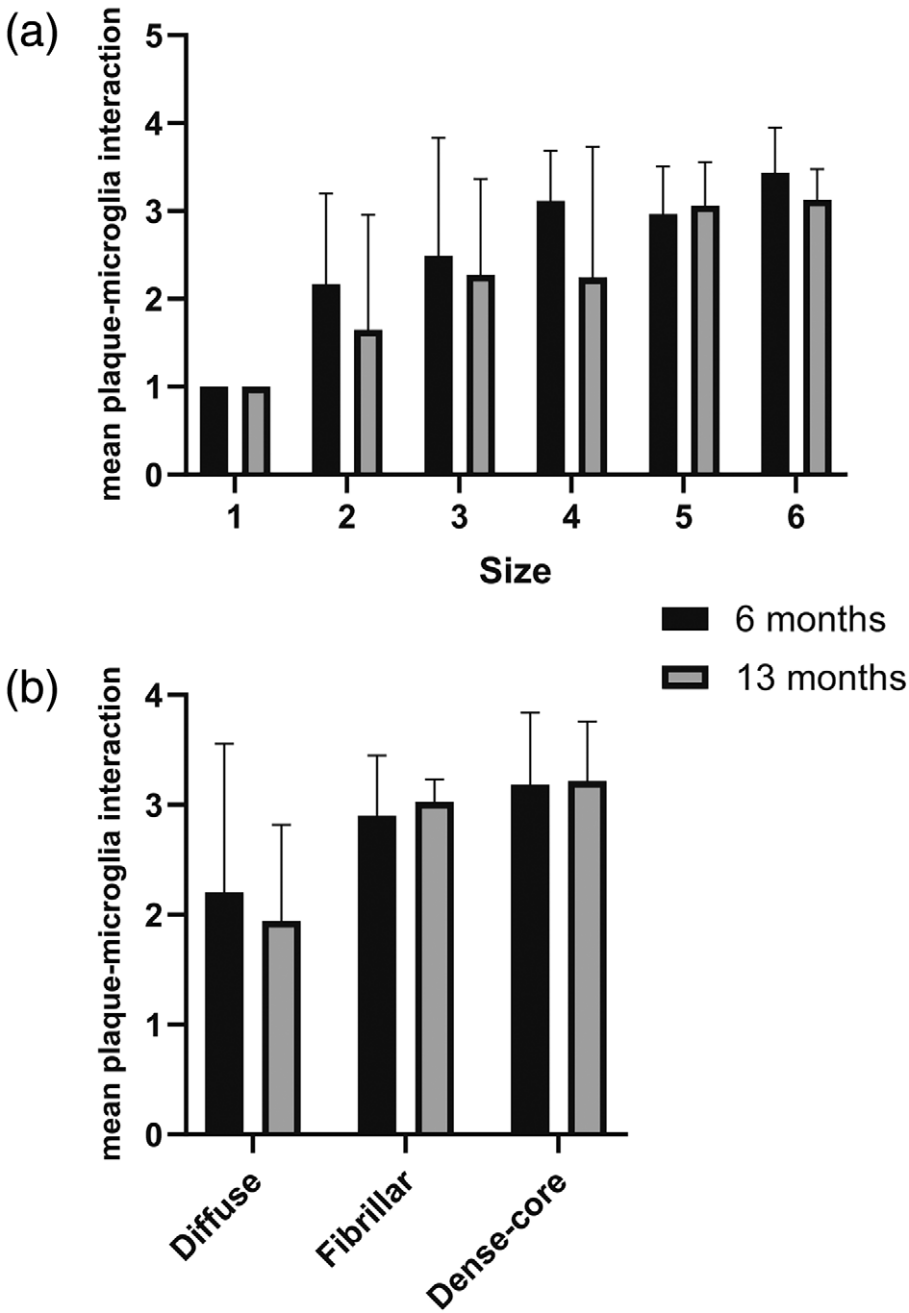
(a) The intensity of microglia–amyloid plaque interaction increases as a function of amyloid plaque size. See Figure 2 for the size categories. Age 6 months: *R* = 0.39, *p* = 0.01, *n* = 44. Age 13 months: *R* = 0.45, *p* < 0.001, *n* = 61, Spearman rho. (b) The microglia–amyloid plaque interaction intensity tends to increase from diffuse plaques toward dense‐core plaques. Age 6 months: *p* = 0.10, *n* = 44; age 13 months: *p* < 0.001, *n* = 60, Kruskal–Wallis H.

The mean interaction category (1–4) of the individual microglia in a cluster around an amyloid plaque is plotted against the amyloid plaque size in Figure 4a. It is obvious that the intensity of the microglia—plaque interaction increases as a function of the amyloid plaque size. This was confirmed by a correlation analysis (6 months: *R* = 0.39, *p* = 0.01, *n* = 44; 13 months: *R* = 0.45, *p* < 0.001, *n* = 61; Spearman rho).

### 3.3 Amyloid plaques with DAPI+ fibrils have more intense interaction with microglia than diffuse plaques

We were further interested in whether the composition of the amyloid plaque relates to the intensity of their interaction with the surrounding microglia. This seemed to be the case as shown in Figure 4b. The microglia—amyloid plaque interaction intensity increased from diffuse plaques toward dense‐core plaques in 13‐month‐old mice (*p* < 0.001, *n* = 60, Kruskal–Wallis H) while a similar trend did not reach significance among the 6‐month‐old mice (*p* = 0.10, *n* = 44; Kruskal–Wallis H).

### 3.4 Microglia insulate with enlarged processes and soma only the dense core

**FIGURE 5:**
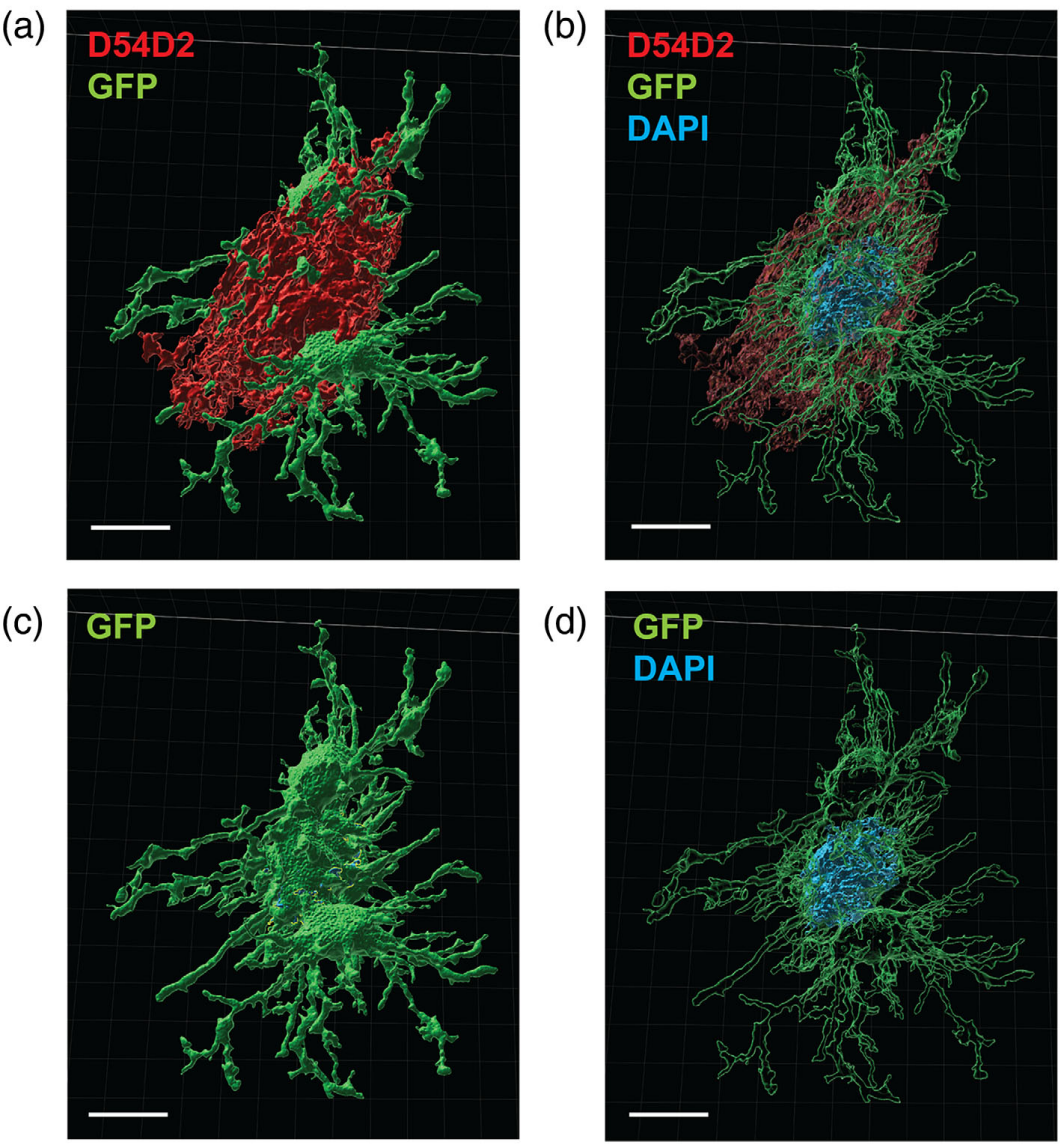
3D IMARIS rendering showing the difference in the microglia interaction with the diffuse plaque shell stained with anti‐Aβ antibody D54D2 and the DAPI+ dense core. (a) Microglia processes (GFP) dive into the loose diffuse amyloid layer (D54D2). (c) Microglia processes (GFP) reach and cover the DAPI+ (blue) core. (b and d) The same images rendered so that the microglia appear transparent to uncover the hidden DAPI+ dense core. Scale bar = 10 μm.

The microglia–plaque interaction categories 1 and 2 involved only thin processes with little contact surface. This matches well with the idea of a surveillance function of homeostatic microglia. Instead, the more intensive microglia–plaque interaction categories involve microglia with enlarged or swollen processes, which is compatible with the idea of reactive microglia. A particularly interesting detail of category 3 interaction is that the microglia appear to encroach their enlarged processes through the diffuse layer of amyloid into the dense plaque core (Figure 3c). To get a better idea of this interaction type, we selected 11 clusters with category 3‐like interacting microglia for a closer examination. With the IMARIS software we defined boundaries of each microglia and the amyloid plaque layers. We also calculated with the MATLAB Surface‐Surface Contact Area XTEnsion for IMARIS software coverage of the diffuse shell and the compact core by microglia processes (Table 1). The analysis clearly showed that microglia processes (or body) cover the DAPI+ dense plaque core much more effectively than the diffuse shell. Figure 5 shows a representative example.

**TABLE 1:** Coverage (%) by combined microglia processes of the D54D2+ diffuse amyloid shell and the DAPI+ dense core in 10 plaques with type 3 interacting microglia.

Mouse	Cluster #	% coverage shell	% coverage core
367	42	11.5	83.9
367	41	11.7	39.5
218	7	12.4	63.5
218	2	15.4	77.6
218	4	17.0	19.5
367	43	17.0	94.3
218	5	17.1	27.9
218	6	18.3	81.5
218	8	20.1	42.8
218	3	25.2	60.0
367	60	30.1	57.0
**Mean**		**17.8**	**58.9**

### 3.5 CD68 immunopositivity is concentrated on the enlarged processes of microglia in contact with the DAPI+ amyloid plaque core

**FIGURE 6:**
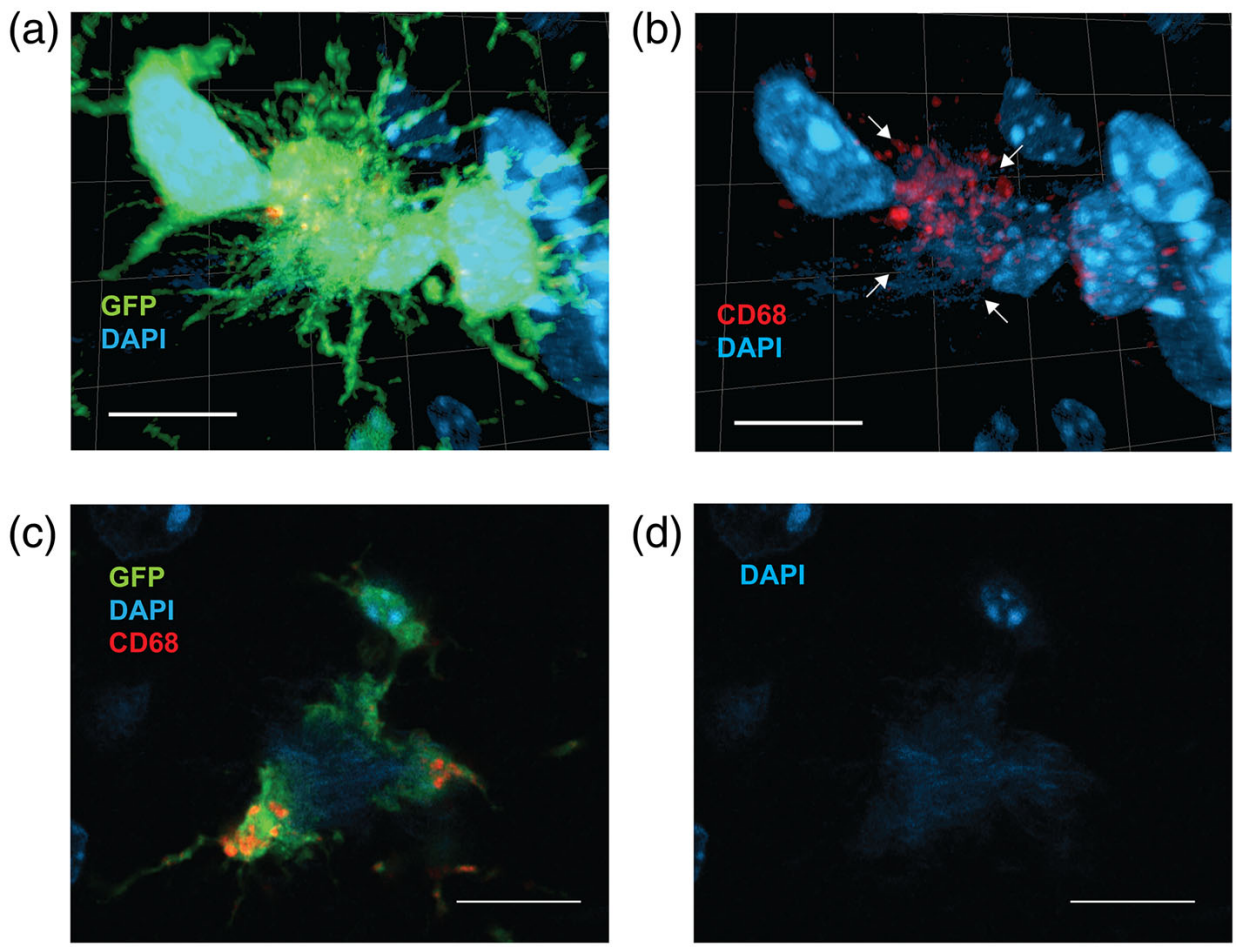
CD68 positivity in the microglia clustered around amyloid plaques. (a) Dense meshwork of processes from two microglia surrounding a DAPI+ fibrillar plaque. (b) The same image without the green channel to better visualize the CD68+ vesicles (red) that in this case are only found in the processes in contact with the fibrillar plaque (DAPI, arrows). (c) A single optical slice through the center of a fibrillar plaque surrounded by three enlarged microglia processes and the soma of one of the microglia. Concentration of CD68+ vesicles is clearly seen in two of the processes. (d) DAPI channels alone to show the extent of the fibrillar amyloid deposit. Scale bar = 10 μm.

The contact of microglia processing with the dense core of the amyloid plaque may serve two functions. First, as suggested by the high coverage, microglia may attempt to insulate the amyloid plaque core from the surrounding neural tissue. Alternatively, the enlarged processes may be engaged with phagocytic activity to remove the amyloid from the tissue and lyse it. To test these hypotheses, we stained the sections with the CD68 antibody, a marker of microglial lysosomes. In general, microglia that clustered around amyloid plaques exhibited significantly stronger CD68 expression that microglia away from amyloid plaques. The CD68 signal was especially concentrated in the enlarged processes that were in contact with the DAPI+ fibrils (Figure 6).

To estimate how common is such CD68 clustering on thickened microglia processes, we examined 25 dense‐core or fibrillar amyloid plaques with type 3 microglia‐plaque interaction. CD68 could be detected around each plaque. In 21 out of 25 plaques, CD68+ vesicles could be localized inside the microglia processes (Figure 6c), and in 22 there was a cluster of CD68+ vesicles perinuclearly in the soma (Figure 6b). These findings indicate that the microglia processes reaching into the plaque core do more than just insulate the amyloid deposition.

### 3.6 Microglia contacting the fibrillar plaque core also contain Aβ + vesicles

**FIGURE 7:**
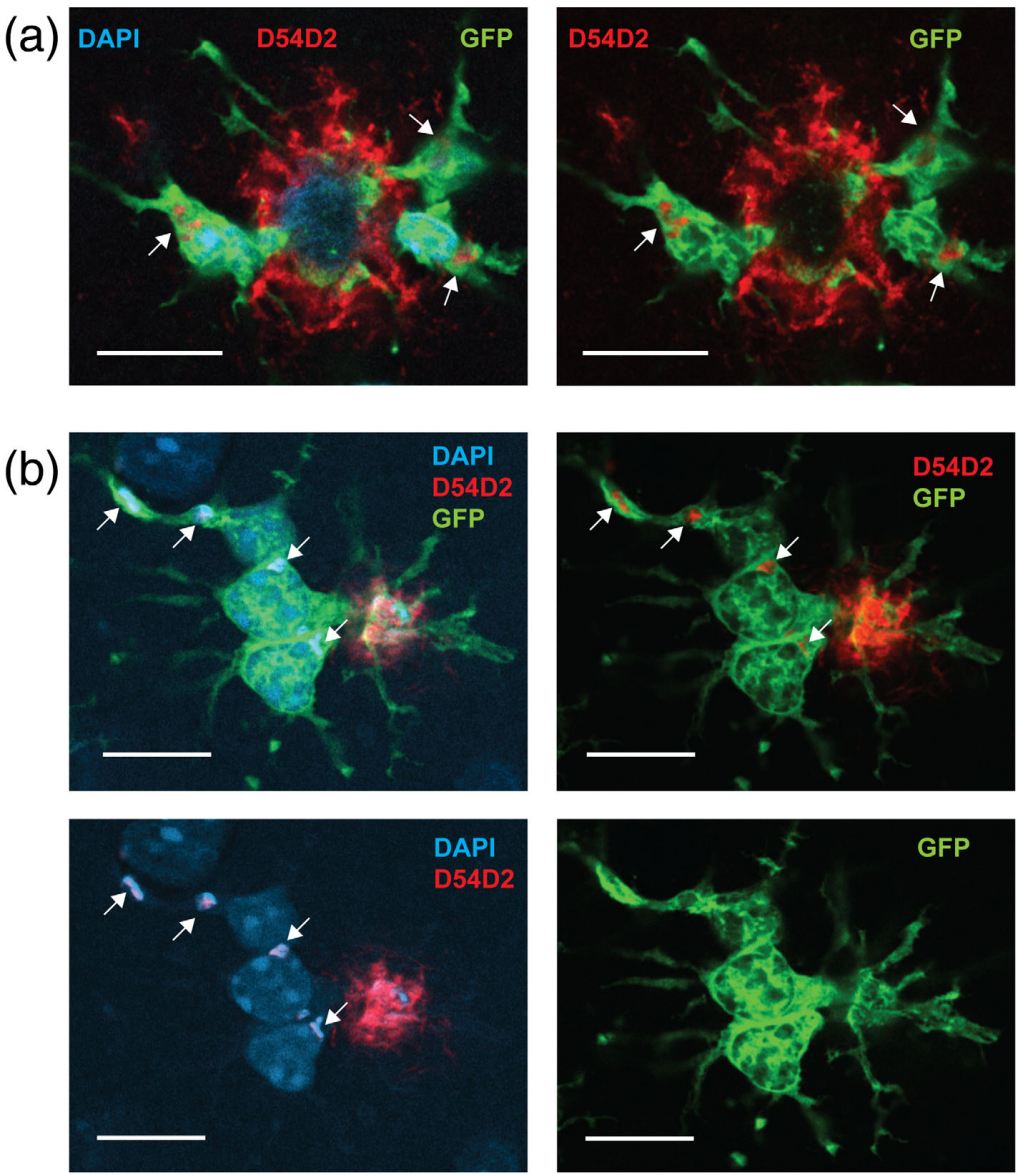
Images of two dense‐core amyloid plaque stained with D54D2 for diffuse amyloid and DAPI for nuclear chromatin and fibrillar amyloid. (a) A single z‐plane image of a plaque surrounded by three microglia, two of which contact the plaque core, while the third one at 4 o'clock is actually on a lower level without any contact with the shown plaque. All three microglia display small D54D2+/DAPI− blobs in the cell body, compatible with perinuclear lysosomes (arrows). (b) A merged z‐stack image of a small dense‐core plaque that is contacted from below by one microglia with a plate‐like extension. The microglia is abutted by two other microglia with no direct contact with the plaque. All three microglia display D54D2+/DAPI+ perinuclear deposits (arrows), presumably in an enlarged lysosome. Scale bar = 10 μm.

Healthy mouse microglia are effective at taking up soluble forms of Aβ through micropinosytosis and fibrillar Aβ with the help of a cell surface innate immune receptor complex (Lee & Landreth, 2010). To see if microglia that contact the fibrillar plaque core with their enlarged processes are engaged with phagocytosis, we stained select microglia of this type with diffuse amyloid staining D54D2 and DAPI for fibrillar amyloid and searched for evidence of amyloid positive vesicles intracellularly. We did find typical perinuclear anti‐Aβ vesicles that were D54D2 positive (Figure 7). Some of them had no DAPI staining (Figure 7a) but others were strongly DAPI positive (Figure 7b) suggesting that they contained fibrillar Aβ. Most likely the DAPI‐negative Aβ found in perinuclear vesicles (probably phagolysosomes) derived from those processes that are in contact with the diffuse part of the amyloid plaque, whereas the ones with fibrillar Aβ may derive from the processes contacting the plaque core.

### 3.7 ApoE connects the thick microglia processes with the amyloid plaque core

If the microglia are primary engaged in phagocyting Aβ deposits in the brain, why do they extend their thick processes through diffuse layer of amyloid and contact the fibrillar amyloid, thus reaching for a hard bite and missing the easy snack? There must be a chemotactic factor guiding this process. One good candidate for such a factor is ApoE that has been shown to facilitate microglia responses to amyloid plaques and direct their conversion into the DAM phenotype (Keren‐Shaul et al., 2017; Liu et al., 2023).

**FIGURE 8:**
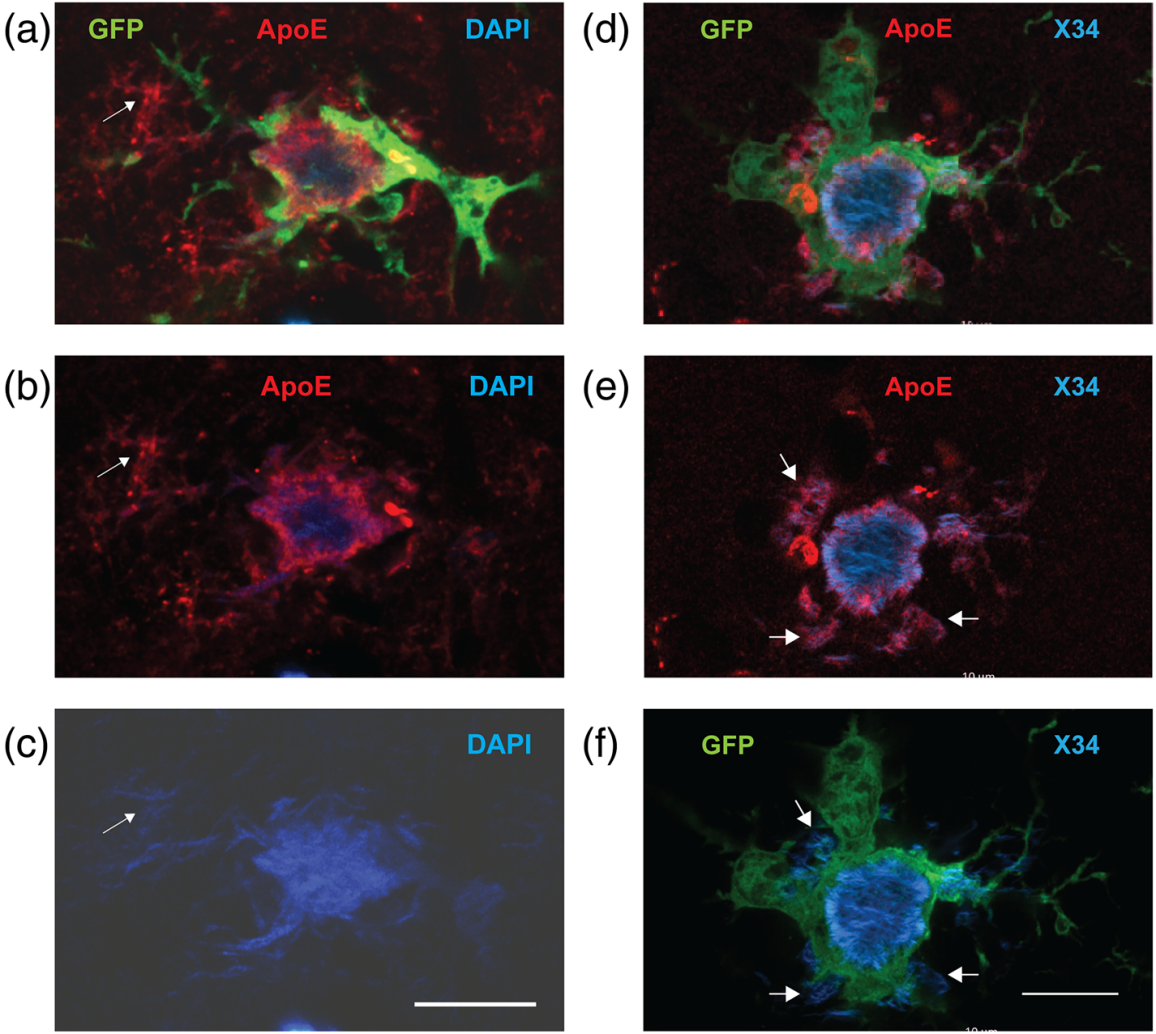
(a) An 18 μm thick single z‐stack plane showing the cross‐section through a small dense‐core plaque with surrounding thick microglia processes (GFP). The DAPI+ plaque core is seen with blue and the ring‐like layer of ApoE around it in red. A fibrillar extension of the amyloid plaque (white arrow) is covered with ApoE as well but is not in direct contact with any microglia process. (b) Red + blue channels, (c) blue (DAPI) channel only. (d) Another dense‐core plaque stained with X34 that highlights amyloid fibrils outside the dense plaque core better than DAPI. (e) ApoE (red) covers all fibrillar Aβ except the dense core. (f) The microglia coverage instead is only partial (white arrows). Scale bar = 10 μm.

To see the spatial relationship between the amyloid plaque, microglia processes and ApoE, we stained sections of APP/PS1 mice expressing GFP in their microglia with anti‐ApoE and DAPI. We focused on dense‐core plaques. In all 14 plaques analyzed with confocal microscopy, we found the same layered organization. The dense DAPI+ core was surrounded by a thin layer of ApoE, which in turn was surrounded by enlarged snowplow‐like microglia processes (Figure 8a–c). Furthermore, ApoE was seen to cover fibrillar amyloid that was not in direct contact with microglia processes (Figure 8a–c, arrows). To study this more quantitatively, we stained sections of the same APP/PS1‐GFP mice with anti‐ApoE and X34 that stains amyloid fibrils outside the plaque core more strongly than DAPI. We sampled 29 well visible dense‐core plaques surrounded by microglial thick processes. Of these, 21 had the ApoE layer between the plaque core and microglia. Further, around 26 out of 29 plaques ApoE was seen to fully cover, and in one case partially cover, fibrillar amyloid outside the dense core (Figure 8d–f, arrows).

The location of ApoE as a thin layer around the dense plaque core between the fibrillar amyloid and the microglia processes is compatible with its function as chemoattractant for the microglial processes. Further, the coating of unorganized DAPI+ amyloid fibrils without microglia contact speaks against the interpretation that the ApoE layer is excreted by the microglial processes.

**FIGURE 9:**
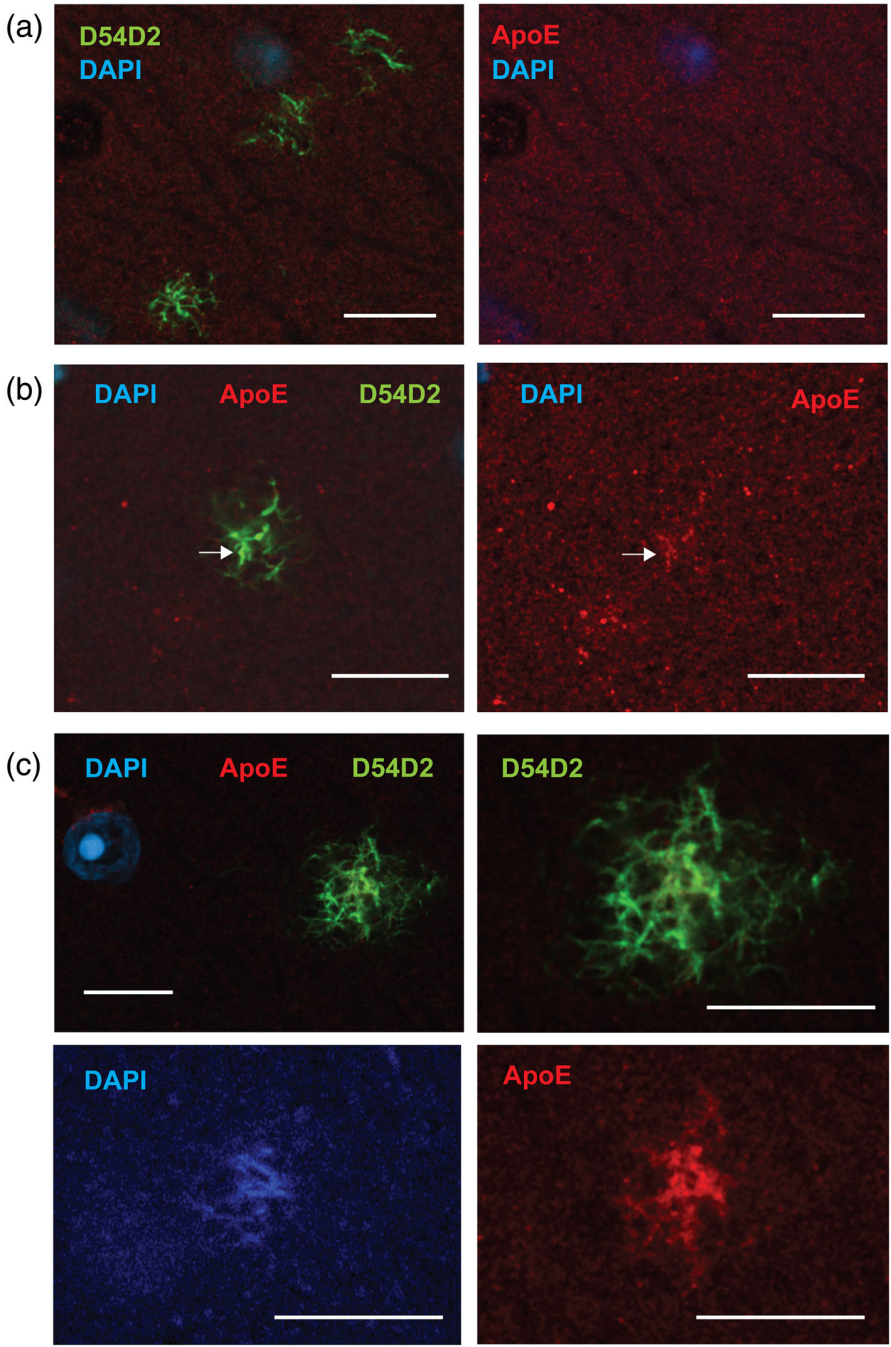
Examples of determinants of ApoE immunopositivity in amyloid plaques. (a) Three small (< 10 μm) diffuse amyloid plaques with no evidence of ApoE immunopositivity. (b) A single diffuse amyloid plaque (<10 μm) with a trace ApoE immunopositivity without overlapping DAPI positivity. (c) A bit larger (> 10 μm) ostensibly diffuse amyloid plaque that is strongly ApoE positive. However, a strong excitation that already saturates the nuclei, reveals a central nidus of fibrillar amyloid that overlaps with the strongest ApoE signal.

Next, we wanted to determine at which stage in the amyloid plaque development ApoE steps in the process. To this end, we stained sections with anti‐Aβ, ApoE and DAPI, and searched for small isolated amyloid plaques with no dense core varying in their diameter. The presence of ApoE positivity strongly correlated with the plaque diameter (Figure 9), so that plaques over 10 μm in diameter were mostly ApoE positive Table 2. Further, among the screened 40 plaques all with discernible DAPI+ fibrils were ApoE positive independent of their diameter (seldom <10 μm, however). The appearance of plaque ApoE immunopositivity thus seems to occur at the stage when the plaque center shows first evidence of fibrillar amyloid and clearly precedes the appearance of DAM‐like microglia interacting with the plaques with their enlarged processes.

**TABLE 2:** Presence of ApoE signal in D54D2 amyloid plaques of different sizes.

Plaque diameter, μm	ApoE presence	Plaque diameter, μm	ApoE presence
4.0	0	10.3	1
4.5	0	10.5	1
4.6	0	10.6	1
6.9	0	10.7	1
7.2	0	10.9	0.5
7.3	0.5	11.0	1
7.3	0	11.3	1
7.4	0	11.5	1
7.7	0	11.8	1
7.7	0	11.9	1
7.9	0	12.4	1
8.0	1	12.4	1
8.2	0	13.0	1
8.3	0	13.4	1
8.6	0.5	13.6	1
8.6	0	15.4	1
9.0	0.5	15.6	1
9.0	0	19.0	1
9.4	0.5		
9.7	0		
9.8	0.5		
9.9	0.5		

*Note*: 0 stands for no ApoE immunopositivity, 0.5 stands for weak positivity, and 1 means clear, bright ApoE immunopositivity.

Abbreviation: ApoE, Apolipoprotein E.

## 4 DISCUSSION

Technical limitations in post‐mortem histopathology make it difficult to visualize the whole cluster of microglia around amyloid deposits in 3D and at the same time reveal structural details of their processes at a high resolution. We managed to solve this challenge by combining several recent technical improvements as described in the Introduction. The GFP expression in the microglia of APP/PS1 transgenic mice enabled homogenous fluorescence in the soma and processes of all microglia. The property of DAPI to stain both fibrillar amyloid and nuclear chromatin with a different intensity and clearly distinct pattern made it possible to see the fibrillar part of amyloid plaques and microglia nuclei in a single color channel. One important advantage of DAPI as a stain for fibrillar amyloid over the standard Thioflavin‐S is that it does not have significant fluorescent in the green range and thereby allows simultaneous use of the GFP label. This study was limited to areas of the hippocampus outside the dense pyramidal and granule cells layers, which greatly helped us to identify the nucleus of each microglia. Otherwise, some enlarged processes could have been misinterpreted as separate cells. Admittedly, our choice to use 100 μm sections was overoptimistic, as the light penetration of our confocal microscope proved to limit to 40 μm from the slice surface. Nevertheless, this range was enough as we were mainly interested in isolated plaques with no satellites.

We divided amyloid plaques into three categories, diffuse, fibrillar, and dense‐core, according to the staining pattern as described earlier (Dickson & Vickers, 2001). We have recently demonstrated that DAPI, which resembles thioflavins in its molecular structure, stains the fibrillar amyloid similarly to Thioflavin‐S or X34 (Mabrouk et al., 2022). These three categories of amyloid plaques most likely represent stages in a continuous process as their prevalence in our sample correlated significantly with the plaque size. Furthermore, in agreement with earlier studies (Bolmont et al., 2008; Jung et al., 2015) we found that the number of microglia around a plaque increases with the plaque size. This relationship appears exponential, which corresponds to the growth of the surface area. Assuming that the amyloid plaque is a sphere, doubling its diameter (from size 1–2 to size 5–6 in Figure 3) results in 2^2 area. Thus, it indicates that the density of microglia per plaque surface area remains by and large constant.

In agreement with an earlier in vivo multiphoton imaging study (Jung et al., 2015), we identified four different types of microglia‐amyloid plaque interactions that can be seen as progressive steps from surveillance to aggressive intervention. These depend both on the plaque size and the composition of the plaques, that is, whether the plaque has a core of fibrillar amyloid or not. The analysis of microglia‐plaque interactions by plaque type revealed a clear distinction between diffuse plaques and plaques with DAPI+ fibrillar component. Diffuse amyloid plaques were surrounded by microglia contacting them with thin processes (categories 1–2), while the thick processes of ‘snowplower’ microglia or microglia body contacts were found around plaques with a fibrillar component. This also means that microglia around diffuse plaques retain their homeostatic morphology with thin, long processes while reactive microglia with ameboid bodies and grossly enlarged processes concentrate around fibrillar or dense‐core plaques as reported earlier (Chen & Colonna, 2021).

Although concentration of microglia around plaques with a dense core in amyloid plaque forming transgenic mice is a common finding, to our knowledge no previous study has tried to determine the exact target of the microglial processes. Our detailed 3D rendering and surface contact analysis utilizing the IMARIS software revealed an unexpected finding. The microglia seemed to extend their processes through the outer diffuse layer of the dense‐core plaque and instead contact the core of the plaque with snowplow‐like end‐feet. The microglia covered a substantially larger proportion (20%–94%) of the plaque core surface than the surface of the diffuse outer layer (11%–30%). First, if the primary response of microglia to amyloid deposition in the brain is to phagocytose the extra accumulated protein, why do they miss the easy prey of diffuse amyloid and concentrate on the hard core? Second, if the microglia primary aim to insulate the amyloid plaque to protect the surrounding neural tissue (Condello et al., 2015; Ulland et al., 2017; Zhao et al., 2017), why do they focus on isolating the inert plaque core rather than the diffuse part that contains the toxic soluble oligomers and protofibrils and is in direct contact with the neural elements?

To assess if the “snowplower” microglia are indeed engaged in phagocytosis, we stained sections with GFP+ microglia with anti‐N terminal Aβ antibody and DAPI, and collected images of these “snowplower” microglia in contact with fibrillar amyloid plaques. We found examples of internalized Aβ immunopositivity in perinuclear vesicles. Some of these vesicles were also DAPI positive, but some were not (Figure 7). It is well acknowledged that microglia can engulf soluble Aβ in phagolysosomes. However, when it comes to fibrillar amyloid, some studies have failed to see intracellular fibrillar amyloid in these vesicles (Liu et al., 2010) while those have been reported by others (Huang et al., 2021; Lee & Landreth, 2010). Two separate uptake mechanisms are involved. Microglia take up soluble Aβ through macropinocytosis, whereas uptake of fibrillar Aβ requires interaction with the cell surface innate immune receptor complex, including among others the TAM receptors (Lee & Landreth, 2010). Based on the fine morphology and orientation of microglia, we suggest that the uptake of soluble Aβ from the diffuse shell of an amyloid plaque happens through the thin microglia processes while the enlarged processes are responsible for phagocytosis of the fibrillar part of the plaque. In support of this view, we saw concentration of CD68 immunoreactivity, a marker of microglia lysosomal vesicles, within the enlarged processes that were in contact with fibrillar plaques (Figure 6).

More recently, the microglia around amyloid plaques have been ascribed a third possible function beside phagocytosis and plaque insulation, namely plaque compaction (Lemke & Huang, 2022). In other words, rather than trying to engulf and degrade dense‐core plaques, the microglia are building them up. This contention is largely based on studies where microglia have been depleted by selective inhibition of their growth factor CSF1 receptor in amyloid plaque forming transgenic mice. The common finding of these studies is reduction of brain overall amyloid load, but also a shift in the proportion between diffuse and dense‐core (compact, fibrillar) plaques such that microglia depleted mice show fewer dense‐core plaques (Spangenberg et al., 2019; Casali et al., 2020). The same observation has been done in mice deficient in microglial TAM‐receptors that are pivotal for phagocytosis of fibrillar amyloid (Huang et al., 2021). Our finding of snowplow‐like enlarged microglia end‐feet full of lysosomes only around amyloid plaques with fibrillar core is compatible also with this idea. It is possible that microglia can shape the early, irregular star‐shape fibrillar amyloid into a more compact and round dense‐core plaque by taking up fibrillar amyloid from the sharp edges, let it polymerize further in the sour lysosomal compartments and release it back into compact layers.

ApoE has been shown to play a key role in amyloid plaque formation. APP or APP/PS1 mice deficient in ApoE show dramatic reduction in the amyloid plaque load in general, and specifically total absence of fibrillar plaques (Bales et al., 1997; Holtzman et al., 2000).

Further, reduction of fibrillar plaques was found also in more aggressive amyloid plaque forming ApoE deficient APP/PS1 mice, including the APdE9 used in the presence study, but not yet total absence of fibrillar plaques (Ulrich et al., 2018). However, a closer examination revealed a fundamental structural difference in fibrillar plaques based on the presence of ApoE. The ApoE knockout mice exhibited plaques with less organized fibrils radiating from the plaque center to all directions (equal to our fibrillar plaques), whereas ApoE wild‐type had well organized dense‐core plaques. Absence of ApoE also resulted in much looser clusters of microglia in the vicinity of amyloid plaques (Ulrich et al., 2018). Furthermore, similarly to amyloid plaque forming APP/PS1 × TREM2−/−mice (Keren‐Shaul et al., 2017; Krasemann et al., 2017; Yuan et al., 2016), these mice lack the DAM‐type gene expression. Collectively, these findings strongly speak for the key role of ApoE in modulating the microglia capable of amyloid plaque compaction. However, microglia selective knockout of ApoE in the 5\u00D7FAD transgenic mouse with amyloid plaque formation did not result in altered amyloid plaque load. The only abnormality in these mice compared to ApoE+/+mice was a larger plaque size in the hippocampus (Henningfield et al., 2022), which speaks for an impaired plaque compaction.

Less is known about the exact location of ApoE around the amyloid plaque. Some studies have reported ApoE co‐localization with microglia (Henningfield et al., 2022, Figure S2g) while another report suggests its source to be astrocytes or neurons (Lau et al., 2023). The standard 2D images of amyloid plaques co‐stained with Iba and Abeta do not allow to see whether ostensibly overlapping staining really means co‐localization. A new finding in this study was that in fully organized dense‐core plaques ApoE immunopositivity was consistently seen as a separate layer between the fibrillar amyloid and the distal processes of the “snowplower” microglia (Figure 8). In addition, ApoE coating was also found on unorganized DAPI‐positive amyloid fibrils. These observations are compatible with the recent evidence that vascular cell adhesion molecule VCAM1 directs microglial Aβ chemotaxis by sensing amyloid plaque‐associated ApoE (Lau et al., 2023), and explain why enlarged microglial processes in our dataset seems to ignore the loose diffuse amyloid plaque shell and orient toward the fibrillar plaque core. In addition, we saw ApoE positivity in the diffuse Aβ‐immunopositive layer of the plaque, but not in significant amounts inside the GFP‐labeled microglia.

We also wanted to assess at which stage of the amyloid plaque development ApoE steps in the process. The smallest diffuse plaques showed no ApoE immunopositivity. These are also plaques that do not attract more microglia interaction than the surveillance type of contact with thin processes. When the plaque diameter reached ~10 μm, also ApoE seemed to be present. Most of the diffuse plaques of this size had a small fibrillar nidus in the center, which was the spot of the highest ApoE immunopositivity (Figure 9). This trend suggests that something in the growing amyloid plaque, most likely Aβ fibrillization, makes ApoE stick to the plaque and start to attract microglia to gather around it. This is also in line with an earlier in vivo multiphoton study suggesting that fibrillar amyloid plaque formation precedes microglia activation (Jung et al., 2015).

These described morphological details in microglia—amyloid plaque interactions shed new light to understand the early steps of Aβ plaque pathology. Combination of the described imaging technique with new therapeutic approaches to modulate microglia responses in AD mouse models will be an interesting avenue in future research.

This study has also some limitations. The method is extremely tedious and time consuming forcing us to focus on only one sex. The rate of amyloid accumulation is faster in female mice (Stephen et al., 2019; Wang et al., 2003) while the microglia coverage of plaques in higher in males (Stephen et al., 2019). We chose female mice as AD is more common in women than in men. It is possible that the coverage of plaque dense core vs. diffuse surface differs between males and females. Furthermore, since DAPI stains nuclear chromatin more strongly than fibrillar amyloid, we needed to restrict our analysis to hippocampal layers free of neuronal somata. It is possible that the microglia–plaque interaction described in this work may be different in neuron dense brain regions with higher overall amyloid load such as the neocortex and subiculum.

## AUTHOR CONTRIBUTIONS

Conceptualization: HT; Formal analysis: MG; Funding acquisition: HT, MG; Investigation: MG; Methology: MG, PM, JC; Supervision: JC, TN, HT; Writing: MG, PM, TN, HT.

## Data Availability

The data that support the findings of this study are available from the corresponding author upon reasonable request.
